# Air Pollution’s Hidden Toll: Links Between Ozone, Particulate Matter, and Adolescent Depression

**DOI:** 10.3390/ijerph21121663

**Published:** 2024-12-13

**Authors:** Megan Waxman, Erika M. Manczak

**Affiliations:** Department of Psychology, University of Denver, Denver, CO 80208, USA

**Keywords:** pollution, ozone, particulate matter, depression, adolescence, mental health

## Abstract

Rising rates of depression among youth present a growing mental health crisis. Despite growing concerns regarding the risks of air pollution exposure on youth mental and physical health, associations between ambient air pollutants and depression have been largely overlooked in youth. In this cross-sectional study, we investigated associations between ozone, particulate matter, and depressive symptoms in adolescents across 224 Colorado census tracts (average age of 14.45 years, 48.8% female, 48.9% of minority race/ethnicity). Students in participating schools reported depressive symptoms and demographic information, and school addresses were used to compute ozone and particulate matter levels per census tract. Possible confounding variables, including sociodemographic and geographic characteristics, were also addressed. Exploratory analyses examined demographic moderators of these associations. Census tracts with higher ozone concentrations had a higher percentage of adolescents experiencing depressive symptoms. Particulate matter did not emerge as a significant predictor of adolescent depressive symptoms. Secondary analyses demonstrated that associations with ozone were moderated by racial/ethnic and gender compositions of census tracts, with stronger effects in census tracts with higher percentages of individuals with marginalized racial/ethnic and gender identities. Ultimately, this project strengthens our understanding of the interplay between air pollution exposures and mental health during adolescence.

## 1. Introduction

Growing rates of depression among adolescents present a major public health concern. Depression is the leading cause of disability worldwide [1], contributing to significant economic and health costs globally [2]. Indeed, experiencing depression during the critical developmental period of adolescence is associated with a myriad of negative outcomes across the lifespan, including long-term mental health, mortality rates, impacts on educational attainment, and financial strain [3,4]. In recent years, significant increases in the rates of depression among adolescents have been reported, with the prevalence of major depressive episodes in adolescents increasing by 7.7% between 2009 and 2019 [5], and 17% of adolescents in the US reporting at least one depressive episode in 2020 [6,7]. Given the importance of mental health in contributing to overall health, furthering our understanding of contributors to adolescent depression is critical to promoting wellbeing across the lifespan.

While psychosocial risk factors for depression across the lifespan are well documented, including social relationships, stress, socioeconomic status, and chronic health conditions [8], one set of risks for depression that has been largely overlooked is air pollution. Outdoor air pollution is recognized as the greatest environmental threat to human health [9]. While air quality reports across the US have shown pollutant concentrations to be trending downwards [10], over 40% of Americans continue to live in areas with unhealthy levels of pollution [11]; this raises concerns regarding the various health problems and environmental impacts linked to pollutant exposure [10]. For example, pollution was estimated to cause 4.2 million premature deaths worldwide in 2019, with strong evidence of associations between air pollution and cardiovascular and respiratory diseases [12,13,14,15]. While air pollutants have been linked to a variety of health problems across the lifespan, the period of adolescence represents a sensitive phase in brain development, suggesting increased susceptibility to the negative impacts of environmental exposure [16]. Indeed, during this critical developmental phase, the natural filters that protect the lungs from inhaled particles are less effective, the breathing rate is increased in proportion to body size, and there is a greater likelihood of spending time outdoors [17,18], indicating that adolescents may be particularly vulnerable to the detrimental effects of air pollution.

Given the links between air pollutants and physical health outcomes, it is plausible to consider the potential impacts of pollution on mental health. Indeed, biological processes that may be influenced by pollutant exposures, including the central nervous system, respiratory system, and systemic inflammation, have also been implicated in the development of depression [19,20]. Regarding the role of pollutants in inflammation, current work has evidenced that exposure to air pollutants can promote inflammatory responses, with chronic exposures leading to systemic inflammation [20,21,22]. The role of inflammation in major depressive disorder (MDD) is well established, with evidence linking elevated levels of inflammatory markers in individuals with depression, and pro-inflammatory markers (e.g., cytokines) implicated in multiple mechanisms involved in depression (e.g., neurotransmitter expression) [22,23]. Taken together, evidence linking pollutant exposures to inflammation and other biological processes implicated in depression provides a potential mechanism whereby pollutant exposures may contribute to these mental health outcomes.

Within the emerging literature on pollution and mental health, two of the most studied pollutants are particulate matter (PM_2.5_), fine inhalable particles with diameters of 2.5 μm or smaller, and ozone (O_3_), a gas created by the product of volatile organic compounds and nitrogen oxides reacting to sunlight. In the current research on adults, depressed mood and an increased risk of depression have been associated with greater PM_2.5_ [24,25,26]. Notably, many of these studies have taken place in cities with notoriously high levels of this pollutant (e.g., [26]), positioning them to pick up on significant associations with particulate matter. The literature examining associations between ozone and depression, however, is less consistent; while some studies have found significant associations between ozone and depressive outcomes (e.g., [27,28]), others have failed to identify significant associations [29,30]. Although the literature on ozone necessitates further investigation, collectively, current research points to a relationship between ambient air pollutants and depression in adults.

While most research to date has concentrated on adults, recent work points to associations between air pollutants and depression in adolescents, a population that is particularly vulnerable to both environmental exposures as well as mental health challenges. For example, several studies investigating associations between PM_2.5_ and adolescent mental health have found that greater pollutant concentrations are linked to both greater self-reported depressive symptoms [31] and risk for a diagnosis of a depressive disorder at age 18 [32]. Taken together, these findings point to particulate matter as potentially contributing to adolescent depression outcomes.

Similarly to research on adults, associations between ozone and depressive symptoms in adolescents are inconsistent. One study found that cumulative ozone exposure in participants’ census tract averaged over 2 years predicted greater increases in adolescent depressive symptoms, and that this association was unique to symptoms of depression versus other psychopathology symptoms [33]. However, a separate study found that long and short-term exposure to ozone at residential addresses, measured as a yearly average or daily average concentrations from 0 to 7 days prior to assessment, were not significantly associated with adolescent depressive symptoms [34]. Indeed, the limited research on this topic necessitates further investigations of ozone and adolescent depressive symptoms to clarify this association.

Existing research has also overlooked factors that may moderate these associations. Individuals with historically marginalized identities are often more likely to experience higher rates of both mental health challenges [35,36] and pollutant exposures [37], suggesting that these individuals may be more adversely affected by pollution. Specifically, rates of depression are higher among females, gender minorities, and individuals who hold racial or ethnic minoritized identities [38,39], with these differences believed to stem from systemic inequities and unequal exposure to stressors. For example, individuals who hold minoritized gender, racial, and ethnic identities have been found to experience greater barriers to accessing mental health care [40,41]. Likewise, the historic redlining of low-income, Black, and Hispanic neighborhoods contributes to disparities in environmental quality and worse environmental outcomes in these communities, including increased PM_2.5_ exposure, compared to non-redlined or majority-White communities [42]. Separately, it is possible that associations between pollution exposure and depression may change as a function of development. Here, one study found associations between depression and ozone to strengthen with age in adolescents [33], but this has not yet been replicated and little is known about other potential moderating effects of race, ethnicity, and gender. Taken together, it is plausible that associations between pollutants and mental health outcomes may be stronger for minoritized groups and older adolescents; such findings could importantly highlight communities that may face a greater risk of adverse mental health outcomes after pollutant exposures.

The current study examined census tract-level associations between ambient air pollutants and depressive symptoms in adolescents using a new tool, the Colorado EnviroScreen, which maps environmental, health, and demographic characteristics across neighborhoods. Census tracts are statistically meaningful and relatively permanent geographic regions in the United States roughly equivalent to the size of a neighborhood (i.e., approximately 4000 individuals), and are frequently used for data analysis and dissemination at the population level [43]. Notably, the incorporation of the EnviroScreen in this project is the first application of this tool to address psychosocial research questions and can serve as an example to advance multi-disciplinary research. To our knowledge, this is the first study to examine these associations in adolescents at a comprehensive state-wide level within the US and the state of Colorado. Indeed, Colorado has significant pollution variability due to its unique terrain and meteorology [44], positioning it as a rich environment in which to investigate associations between air pollution and health outcomes in the United States. This variability in air pollution is similar to other global locations, where cities such as Beijing [45] also contend with pollution influenced by their distinct landscapes and weather patterns, underscoring the need for studies that address the interplay between local environments and health risks worldwide. In the current study, we explored associations between two measures of air pollutants and depressive symptoms in adolescents. Furthermore, given the limitations inherent in correlational studies, it is important to determine whether other factors confound previously observed associations between air pollution and mental health outcomes. We hypothesized that greater levels of ozone and particulate matter exposure would be associated with a higher percentage of adolescents within census tracts reporting depressive symptoms, above and beyond the associations due to relevant geographic and sociodemographic variables. Additionally, in secondary exploratory analyses, the current study investigated demographic factors that may moderate these associations, including census tracts’ composition of race/ethnicity, age, and gender identity. Taken together, this project sought to further our understanding of associations between ambient air pollutants and depressive symptoms in adolescents, as well as identify groups that may face a greater risk of adverse outcomes, ultimately aiming to inform interventions and preventative efforts to promote health across the lifespan.

## 2. Method

### 2.1. Study Overview

Psychosocial data were obtained through the 2021 Health Kids Colorado Survey (HKCS), a survey of the health and wellbeing of high school and middle school students in Colorado [46], the design of which is described in further detail below. Individual data were aggregated based on school location to calculate the percentages or means of each variable per census tract. Environmental data were obtained from the Colorado EnviroScreen [47], an online tool created by the Colorado Department of Public Health & Environment (CDPHE). Census tract-level data from HKCS were merged with the EnviroScreen dataset to create estimates of air pollutants per census tract, reflecting school neighborhoods.

Several other publicly available surveys were used to incorporate additional variables of interest, which are elaborated in the measures section below. The United States Geological Survey (USGS) was used to assess mean elevation per census tract [48]. Additionally, the American Community Survey, a yearly survey that provides information about the US population and housing characteristics, was used to assess population density per Colorado census tract [49].

### 2.2. Participants

All public high schools and middle schools were eligible to participate in the Healthy Kids Colorado Survey by the CDPHE. Up to 7 schools per region, out of 21 health regions (i.e., aggregations of counties developed by the CDPHE), were sampled to take part in the survey; 109 sampled middle and high schools, which included 52,799 sampled students, participated. Schools that were not sampled were eligible to participate in the HKCS by voluntarily opting in. Including both sampled and opt-in schools, a total of 340 public schools and 106,799 students participated. The HKCS used a probabilistic, two-stage clustered sampling design with stratification to ensure the sample was representative of the target population. Indeed, the design also incorporated measures (e.g., census participation and oversampling) to enhance data precision. Survey and recruitment approval was obtained by the Colorado Multiple Institutional Review Board, the Educational Data Advisory Committee, and the HKCS Steering Committee, and informed consent was obtained from all participants in the study. Given the overlapping addresses of middle schools and high schools within the same building, location, or neighborhood, 224 unique census tracts were identified with addresses from 340 schools.

Data were collected for 106,799 students who participated in the HKCS through their middle or high school (48.8% female; 44.1% male; 7.1% nonbinary). The current study size was based on all respondents to the survey. Participating adolescents were on average 14.45 years old (SD = 1.912); 51.1% identified as White, 19.5% identified as Hispanic or Latinx, 18.4% identified as Multiracial, 3.3% identified as Black or African American, 2.3% identified as Asian, 1.5% identified as American Indian or Alaskan Native, 0.4% identified as Middle Eastern, North African, or Arab, and 0.2% identified as Native Hawaiian or Pacific Islander. The Colorado 2020 Census reports similar percentages for the state of Colorado, including 70.7% White, 21.9% Hispanic or Latinx, 12.3% Multiracial, 8% Other Race, 4.1% Black or African American, 3.5% Asian, 1.3% American Indian and Alaska Native, and 0.2% Native Hawaiian or Pacific Islander [50].

## 3. Measures

### 3.1. Particulate Matter (PM_2.5_)

The Colorado EnviroScreen provides 24 h average PM_2.5_ concentrations (μg/m^3^) calculated from 2017. To create these estimates, the EnviroScreen inputted PM_2.5_ air quality monitoring data from State and Local Air Monitoring Stations (SLAMSs) and numerical output from the Community Multiscale Air Quality (CMAQ) model into a Bayesian Space-time Downscaling Fusion Model to predict 2017 concentrations at the 2010 US census tract centroids encompassed by the CMAQ modeling domain [51].

### 3.2. Ozone (O_3_)

The EnviroScreen provides daily maximum, 8 h average estimates of ozone (ppb) averaged from 2017. Daily 8 h maximum ozone concentrations were measured from multiple monitoring locations that subsequently reported values to the Environmental Protection Agency (EPA). As described above, the EnviroScreen used a downscaler model to combine monitoring data from SLAMS and CMAQ outputs to predict daily air pollution at the center point of a census tract [52].

### 3.3. Depression

Symptoms of depression were measured with the HKCS using responses to the following question, “During the past 12 months, did you ever feel so sad or hopeless almost every day for two weeks or more in a row that you stopped doing some usual activities?”. Response options were dichotomous (yes or no). Individual responses were aggregated to construct a variable representing the percent of respondents experiencing depressive symptoms per census tract. Although single-item measures are less psychometrically robust, previous work has demonstrated that when clinical interviews or symptom scales are not feasible, single-item questions are a useful alternative. For example, a study examining agreement between a single-item measure of depression and a depression scale found that the single-item measure demonstrated moderate levels of sensitivity and specificity against the depression scale [53]. Separately, a study that developed ultra-brief single-item depression assessments found evidence of high reliability and validity of single-item measures of depression symptom severity, such that single-item questions accurately discriminated between depressed patients and non-depressed patients [54]. Thus, single-item measures of depression are useful and psychometrically valid alternatives for identifying who may be at risk for depression when clinical interviews or longer measures are not possible.

### 3.4. Demographic Variables

The HKCS measured age [10, 11, 12, 13, 14, 15, 16, 17, 18], gender identity (female, male, nonbinary, questioning, other), and racial/ethnic identity (American Indian or Alaska Native; Black or African American; East or Southeast Asian; Hispanic or Latinx; Middle Eastern, North African, or Arab; Native Hawaiian or Pacific Islander; South Asian; White; or Multiracial). At the participant level, data were initially collected with racial or ethnic identity and gender identity as nominal (categorical) variables. Individual responses were then recoded and aggregated to construct variables indicating percent gender, percent racial and ethnic group, and average age per census tract.

The Colorado EnviroScreen provides the percentages of households with low income per census tract, which is defined by the percent of a census tract’s population living in households where the household income is less than or equal to twice the federal poverty level. This measure of low income is used to identify disproportionately impacted communities, which are defined by the Environmental Justice Act as being any census block group with 40% or more of the population living at or below twice the federal poverty level [55].

### 3.5. Asthma

Current evidence links asthma with increased rates of depression in adolescents [56], as well as increased air pollution exposure and poorer health outcomes [57,58]. Given the potential of asthma to confound the relationship between air pollution and mental health, asthma was assessed with the HKCS using the following question, “Has a doctor or nurse ever told you that you have asthma?”. Response options included yes, no, or not sure. Individual responses were aggregated to construct a variable indicating percent asthma per census tract. Responses of “not sure”, which comprised 7.74% of responses, were treated as missing data, given that treating them as “yes” or “no” responses may have caused an over or underestimation of percent asthma per census tract.

### 3.6. Elevation

Elevation is the mean altitude in meters per census tract. High-altitude regions have poorer air quality, with increasing elevation leading to more intense solar radiation and therefore greater ozone air pollution [59]. Additionally, given significant associations between depressed mood and living at higher altitudes [60], it is important to consider how higher altitude communities may likewise confound the relationship between greater pollutant exposure and mental health psychopathology.

Data were collected by the USGS, a scientific agency that studies the landscape, natural resources, and hazards in the US. Mean elevation per census tract was calculated using a Digital Elevation Model in ArcGIS, a geographic information system. Given its mountainous landscape, elevation across the state of Colorado ranges from 3315 to 14,433 feet, with different census tract neighborhoods sitting at different altitudes [61].

### 3.7. Population Density

Population density is a calculation of the number of people per square mile in each census tract neighborhood area. Greater population density is associated with increased air pollution, including PM and O_3_ [62]. Separately, a systematic review found significant associations between higher population density and depressive mood [63]. Accordingly, it is possible that population density may account for, and thus confound, the association between air pollutants and mental health outcomes; therefore, population density was included as reported by the 2015–2019 American Community Survey.

### 3.8. Transparency and Openness

The authors report no potential conflicts of interest. This study’s design and its analysis were not preregistered. All data have been made publicly available by the CDPHE and can be accessed at https://teeo-cdphe.shinyapps.io/COEnviroScreen_English/ (version 1.0) and https://cdphe.colorado.gov/healthy-kids-colorado-survey-dashboard (survey year 2021). Data were analyzed using IBM SPSS Statistics for Windows, version 28.0 [64]. Materials and analysis code for this study can be shared upon request.

## 4. Analyses

### Analytic Plan

To first examine the characteristics of the study sample, Pearson correlations were run between primary variables. Next, linear regression models were run to test whether associations between pollutants and percentage of depressive symptoms were significant after accounting for relevant demographic and geographic variables, which included age (average age of student respondents per census tract), race/ethnicity (percentages of census tract endorsing each identity, respectively), gender (percentages of census tract endorsing male, female, and nonbinary identities, respectively), economic status (percentage of households with low income per census tract), population density, elevation, and physical health risk (percentage of residents with asthma). Finally, for associations that were significant, exploratory interaction analyses were conducted to assess whether associations differed by average age, percentage of racial/ethnic minority individuals, or percentage of female and nonbinary individuals. (Here, data related to gender and racial identities were recoded into binary variables representing percentage male [versus female and nonbinary] and percentage white [versus all other identities] for moderation analyses). Demographic variables, air pollution variables, and relevant covariates were centered before calculating the interaction term.

## 5. Results

### 5.1. Characteristics of the Study Sample

Descriptive statistics for study variables are presented in Table 1. Associations between census tract characteristics and air pollutants are presented in Table 2. The distributions of all variables were tested for possible violation of normality, and none were determined.

Census tracts with a greater percentage of low-income residents and higher rates of asthma had lower ozone concentrations. Further, census tracts with greater percentages of African American, Hispanic, and Multiracial respondents had lower ozone exposures. In census tracts with a greater percentage of White respondents, there was also greater ozone. Furthermore, in census tracts with lower population density and greater elevation, there was more ozone, whereas in tracts with greater population density and less elevation, there was more particulate matter. The fact that census tracts at higher elevations had greater ozone and more densely populated areas had greater particulate matter likely contributed to the significant negative association between ozone and particulate matter.

### 5.2. Associations with Depressive Symptoms

**Figure 1:**
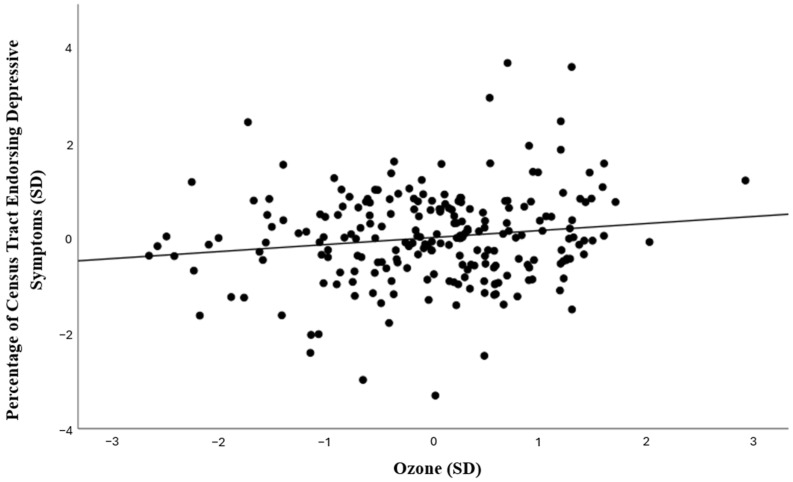
Plot of standardized residuals of percentage of depressive symptoms and ozone levels per census tract, removing the effects of covariates (race, ethnicity, gender identity, age, asthma, low income, population density, elevation), and including a line of best fit.

To estimate associations between depressive symptoms and ozone concentrations, a linear regression model was conducted. As shown in Table 3, the association between ozone and depressive symptoms was significant, above and beyond contributions of relevant covariates (*b* = 0.19, *SE* = 0.37, *t* = 2.14, *p* = 0.034; see Figure 1), such that census tracts with higher ozone had larger percentages of students who reported depressive symptoms. In addition, higher percentages of students identifying as nonbinary for gender identity, American Indian or Alaskan Native, Hispanic, or Multiracial, who had asthma, were low income, or had an older average age were significantly associated with larger percentages of students endorsing depressive symptoms, while census tracts with more students identifying as Native Hawaiian or Pacific Islander had a smaller percentage of students who reported symptoms (See Table 3).

A parallel approach was next used to examine associations with particulate matter. Particulate matter was not significantly associated with depressive symptoms when examined with covariates (*b* = −0.06, *SE* = 0.50, *t* = −0.63, *p* = 0.532; see Table 3). The pattern of significance among covariates remained the same (See Table 3). An exploratory model that simultaneously included ozone and particulate matter showed similar results, where ozone significantly contributing to depressive symptoms and particulate matter was not a significant predictor.

### 5.3. Secondary Analyses: Interactions with Demographic Variables

To probe potential moderation by gender, gender identities were recoded to create a single code representing the percent of the census tract’s respondents reporting being male vs. female or nonbinary. When examining the interaction between gender and ozone, the association with depressive symptoms strengthened as percent male participants decreased (i.e., female and nonbinary participants increased) *(b_ozone_* = 0.19, *SE* = 0.39, *t* = 2.04, *p* = 0.043; *b_gender_* = −0.09, *SE* = 0.07, *t* = −1.61, *p* = 0.108; *b_ozoneXgender_* = 0.13, *SE* = 0.04, *t* = −2.25, *p =* 0.026). Tests of simple slopes revealed that associations at 1 standard deviation above the mean on percent male were not significant; however, at one standard deviation below and at the mean, there were significant associations between ozone and depressive symptoms per census tract (1 SD below mean, *b* = 1.12, *SE* = 0.41, *t* = 2.72, *p* = 0.01; at mean, *b* = 0.76, *SE* = 0.39, *t* = 1.94, *p* = 0.05; see Table 4 for complete models of moderation analyses).

Likewise, to probe potential moderation by race and ethnicity, racial/ethnic identities were recoded into a single code representing the percent of the census tract’s respondents reporting a White racial identity versus all other racial and ethnic identities. Probing the interaction between race and ozone, it was found that the association with depressive symptoms likewise strengthened as the percent of Non-White participants per census tract increased (*b_ozone_ =* 0.18, *SE* = 0.37, *t* = 1.95, *p* = 0.053; *b_race_* = 0.30, *SE =* 0.02, *t* = 4.76, *p <* 0.001; *b_ozoneXrace_* = 0.11, *SE* = 0.01, *t* = 2.06, *p* = 0.041). Simple slope tests revealed that associations at 1 standard deviation below the mean and at the mean on percent Non-White were not significant; however, associations at 1 standard deviation above the mean were significant (*b* = 1.34, *SE* = 0.49, *t* = 2.73, *p* = 0.01; see Table 4).

Finally, investigating the interaction between age and ozone indicated that the association between ozone and depressive symptoms did not significantly change as a function of average age (*b_ozone_* = 0.18, *SE* = 0.38, *t* = 1.97, *p* = 0.05; *b_age_* = 0.55, *SE* = 0.32, *t =* 9.03, *p =* 0.000; *b_ozoneXage_* = 0.04, *SE* = 0.20, *t* = 0.73, *p* = 0.467).

## 6. Discussion

The results of this study indicate that adolescents who live in census tracts with higher ozone concentrations may be at greater risk of experiencing depressive symptoms, above and beyond the contributions of sociodemographic and geographic factors that may account for this association. Additionally, exploratory analyses found that associations between adolescent depressive symptoms and ozone were moderated by race and gender, but not by age. Contrary to hypotheses, parallel findings were not significant when considering associations between particulate matter and adolescent depression. This is the first study to examine these associations in adolescents at a comprehensive state-wide level within the US and the state of Colorado, a state with significant variability in air quality, and one of the first to consider multiple pollutant types and to test moderators of these associations. In doing so, this study adds to the growing literature linking pollutant exposures and adolescent mental health outcomes, highlighting the importance of considering the physical environment in understanding growing rates of depression during this vulnerable developmental period. Importantly, the implications of these findings may inform global health promotion strategies aimed at mitigating the impact of environmental exposures on mental health outcomes.

These findings strengthen the existing research on associations between ozone levels and adolescent depression. The current literature linking depression and ozone in adolescents has been both limited and inconsistent, marking the current study as an important addition in establishing ozone as a potential contributor to the risk of adolescent depression. Indeed, there may be differences in the measurement of depression or failure to comprehensively consider covariates that have contributed to conflicting findings across existing studies. Additionally, the current study sample may have been better positioned to detect associations with ozone, given the high concentrations in Colorado, compared to previous work in locations with lower base rates of ozone that did not find significant associations [34], highlighting the importance of investigating these associations across diverse environmental contexts globally. Although we were not able to directly assess the mechanisms connecting ozone to depressive symptoms, we hypothesize that biological processes, such as systematic inflammation [65], brain signaling [66], and neurotransmitter expression [67] are implicated, providing important directions for future research.

Contrary to hypotheses, particulate matter did not emerge as a significant predictor of adolescent depressive symptoms in this sample. Meanwhile, some prior work, though limited, has identified associations between particulate matter and adolescent depressive symptoms (e.g., [31]) other studies have similarly failed to find significant associations [68]. Indeed, current evidence differs greatly with regard to quality and methodology [69], and the existing literature on adolescents remains sparce. Additionally, the current project may have been better poised to identify associations with ozone exposures versus particulate matter. For example, trends in PM_2.5_ concentrations over the last 20 years show that concentrations are generally low across Colorado [70], whereas the state has high geographic variability in ozone, given its elevation, terrain, and meteorology [71], making it potentially better suited to pick up meaning variability with ozone relative to particulate matter. Indeed, the current project only probed linear relationships between pollutants and depression; thus, future work may explore the possibility of a nonlinear relationship between pollutants and depressive symptoms.

Given significant associations between ozone and depressive symptoms, exploratory analyses probed moderations by racial and ethnic identity, gender identity, and age. Associations were stronger for census tracts with a greater percentage of participants identifying as Non-White and non-male (i.e., female and nonbinary), offering initial evidence suggesting that factors associated with these historically marginalized identities may contribute to increased susceptibility to the impacts of ozone exposure on depressive symptoms. Future research is needed to clarify which factors may be driving these associations, such as discrimination and stigma, history of redlining, reduced access to mental health or community resources, etc. While Manczak et al. [33] found associations between ozone and depressive symptoms strengthened with age, this was likely due to probing depressive symptoms beginning with prepubertal youth (9–13 at baseline), as depressive symptoms are likely to increase in adolescence; in contrast, participants in the current study were above prepubertal age on average (mean = 14.45). Taken together, these findings represent important information regarding groups that may be at greater risk of adverse outcomes after pollutant exposures.

The finding that census tracts with higher ozone also had more adolescents reporting depressive symptoms, with stronger associations for historically marginalized groups, has several notable implications. First, the results are consistent with the possibility that ozone is a significant contributor to adolescent depression, which may improve our understanding of the etiology of depression and the growing rates among youth. Additionally, this highlights the importance of considering environmental exposures for both physical health and mental health outcomes. In broadening our understanding of contributors to mental health outcomes among youth, this work also lends support to the Ecological Theory of Development [72], which emphasizes how development is influenced by a series of interconnected environmental systems, including our physical environment and the policies that monitor it. Taken together, considering a wide variety of individual, social, and environmental factors is important in understanding the etiology of youth mental health outcomes.

If replicated, the current findings also have implications for family and school health decision making and larger governmental policy across the world. While air pollution is ubiquitous, there are several ways in which both families and schools can help minimize exposure. For example, the Air Quality Index (AQI) is a tool developed by the Environmental Protection Agency (EPA) to monitor air pollutant levels for five major air pollutants. Using the AQI, AirNow is a tool that reports precise air pollutant estimates to help users decide whether it is safe to enjoy outdoor activities [73]. Families and schools could utilize available air pollutant reporting resources to make informed decisions about spending time outside during high exposure days and may consider taking extra precautions such as wearing a mask or moving outdoor activities indoors when needed. Importantly, most children spend a majority of their time during the day (i.e., peak ozone hours) at school, highlighting that both families and school policy makers should be involved in minimizing pollutant exposures.

At the government level, the current findings reiterate that policy makers should prioritize ozone violations as a critical issue and make the necessary changes to ensure that pollutant levels are safe and remain below national standards and align with global environmental goals. This may include funding renewable energy projects and enacting environmentally supportive legislation akin to the Clean Air Act in the United States [74] to increase penalties for lapses in compliance with EPA standards and create more stringent guidelines for pollutant emissions. On a global scale, pollution reduction strategies involve greater use of renewable energy, reducing or eliminating use of solid fossil fuels, and increasing use of low-pollution vehicles [75]. Indeed, international collaboration is critical to establish emission standards, promote use of renewable energy, and address air quality concerns worldwide.

There are several limitations of the current project. First, this study examines associations at a census tract level rather than at an individual level. While this can provide important information about community risk, there is likely to be considerable heterogeneity of exposure to pollution among people living within the same census tract. Indeed, reliance on census track level data means that the current project lacks precision in measuring personal air pollution exposure (which might better account for individual differences in time spent outdoors, travel, etc.). Another limitation is the lack of temporal precision in air pollution estimates. The Colorado EnviroScreen provides yearly averages of air pollutant levels, and thus does not disaggregate by seasonal variations in air pollutant exposure. For example, increases in ozone during summer in Colorado are well documented, due to a combination of hot and sunny weather conditions which facilitate the chemical reactions that produce ozone, as well as increased wildfires and vehicle emissions [44,71,76,77]. Thus, it is possible that associations between mental health and air pollutants differ by season, given greater exposures to different pollutants across the year. Additionally, the current project could not account for regional pollution events, which may affect yearly estimates and differences in personal air pollution exposure. An additional limitation is the reliance on brief, self-reported assessments of depressive symptoms. While in-depth, clinician administered measures are generally more sensitive to symptom severity and presentation, they are often not feasible in large, state-wide school-based assessments, such as were examined in the current study. Additionally, although the current project covaried for asthma as a measure of physical health and inflammation, the HKCS did not collect data on the presence of specific chronic diseases or health conditions, or subjective/perceived health, which can also potentially impact the association between air pollution and depression. Importantly, school environment and learning atmospheres may also impact adolescents’ mental health, which could not be accounted for in the current project. Future work may seek to better understand the potential role of school culture in these associations. Finally, while all available school data on Colorado were used to estimate depressive symptoms per census tract, there may have been systematic reasons why certain students in the state did not complete the HKCS, which were not accounted for in the current analyses.

## 7. Conclusions

Ultimately, the current project represents an important addition to the literature in establishing associations between ozone exposures and adolescent depression, providing several directions for future research. First, it will be important for future work to clarify the mechanisms underlying associations between pollutant exposures and mental health outcomes in youth. Specifically, studies employing longitudinal measurements of environmental exposures, biological pathways (e.g., inflammation), and mental health may help to clarify biological mechanisms underlying this association. Understanding how these associations vary based on neighborhood and family factors may explain increased risk among historically marginalized groups. Additionally, future work should consider factors that may protect against or increase the risk of the adverse effects of pollutant exposure on mental health outcomes, such as social support or comorbid mental or physical health conditions. Additional research may also seek to consider the effects of both indoor and outdoor pollutants, which would provide a more precise estimate of individual exposures. Ultimately, despite its limitations and remaining questions, this study provides evidence for the association between ozone and adolescent depressive symptoms, highlighting the role of physical environmental exposures in adolescent mental health risk.

## Figures and Tables

**Table 1:** Descriptive statistics for study variables.

Variable	M	SD	Min	Max
1. Depression	37.58%	7.22	19.23	65.59
2. Ozone (ppb)	45.47	1.75	40.20	49.63
3. PM_2.5_ (μg/m^3^)	6.27	1.41	4.00	8.78
4. Female	44.15%	6.23	30.98	90.91
5. Male	48.72%	5.82	9.09	66.67
6. Nonbinary	7.13%	3.13	0.00	19.30
7. Age (Mean)	14.21	1.40	11.81	17.56
8. Low Income (Composite out of 1)	0.29	0.15	0.04	0.70
9. Asthma	19.29%	5.08	5.60	50.00
10. American Indian or Alaskan Native	1.75%	1.76	0.00	16.60
11. African American	3.44%	5.65	0.00	57.14
12. Asian	1.76%	2.14	0.00	16.72
13. Hispanic	21.71%	17.83	0.00	77.04
14. Middle Eastern, North African, Arab	0.35%	0.63	0.00	4.36
15. Native Hawaiian or Pacific Islander	0.23%	0.38	0.00	2.86
16. White	48.16%	22.28	0.00	93.33
17. Multiracial	18.89%	6.04	5.085	34.84
18. Population Density (People/square mi)	2461.06	2564.74	0.80	12,758.00
19. Elevation (m)	1890.68	511.83	1107.6	3497.25

**Table 2:** Zero-order correlations.

	2	3	4	5	6	7	8
1. Depression	−0.12	−0.01	0.43 **	0.31 **	0.42 **	0.14 *	−0.13
2. Ozone		−0.41 **	−0.03	−0.29 **	−0.25 **	−0.25 **	0.79 **
3. PM_2.5_			0.06	−0.25 **	0.04	0.65 **	−0.61 **
4. Age				0.03	0.35 **	0.03	−0.05
5. Low Income					0.19 **	0.13 *	−0.09
6. Asthma						0.07	−0.24 **
7. Population Density							−0.44 **
8. Elevation							

* Correlation is significant at the 0.05 level (2-tailed). ** Correlation is significant at the 0.01 level (2-tailed).

**Table 3:** Multiple regression models for depression by pollutant type.

Predictor	R	R^2^	F	Sig.	*b*	*SE*	*t*	*p*	VIF	95% CI	
Model Ozone	0.71	0.51	14.36	<0.001							
Ozone					0.19	0.37	2.14	0.034	3.43	0.06	1.52
Nonbinary					0.34	0.14	5.60	0.000	1.54	0.50	1.05
Female					0.01	0.06	0.25	0.800	1.32	−0.11	0.14
American Indian or Alaskan Native					0.11	0.22	2.10	0.037	1.25	0.03	0.91
African American					0.05	0.07	0.79	0.432	1.42	−0.09	0.20
Hispanic					0.20	0.03	3.28	0.001	1.63	0.03	0.13
Asian					−0.08	0.21	−1.32	0.188	1.70	−0.70	0.14
Middle Eastern, North African, or Arab					0.00	0.71	0.01	0.990	1.62	−1.39	1.40
Native Hawaiian or Pacific Islander					−0.12	1.00	−2.21	0.028	1.16	−4.19	−0.24
Multiracial					0.24	0.07	4.06	0.000	1.51	0.15	0.43
Asthma					0.13	0.08	2.10	0.037	1.49	0.01	0.34
Age					0.55	0.32	9.03	0.000	1.59	2.22	3.47
Percent low income					0.13	3.17	1.97	0.050	1.80	0.01	12.52
Population density					−0.06	0.00	−0.92	0.357	1.83	0.00	0.00
Elevation					−0.13	0.00	−1.43	0.155	3.83	0.00	0.00
Model PM_2.5_	0.71	0.50	13.80	<0.001							
Fine particle pollution (μg/m^3^)					−0.06	0.50	−0.63	0.532	3.91	−1.29	0.67
Nonbinary					0.34	0.14	5.57	0.000	1.54	0.50	1.06
Female					0.01	0.07	0.22	0.830	1.34	−0.12	0.14
American Indian or Alaskan Native					0.11	0.23	2.00	0.047	1.25	0.01	0.89
African American					0.03	0.07	0.55	0.580	1.42	−0.11	0.19
Hispanic					0.20	0.03	3.09	0.002	1.74	0.03	0.13
Asian					−0.06	0.22	−0.92	0.358	1.82	−0.65	0.23
Middle Eastern, North African, or Arab					0.00	0.71	−0.05	0.960	1.62	−1.45	1.37
Native Hawaiian or Pacific Islander					−0.11	1.01	−2.00	0.047	1.15	−4.00	−0.03
Multiracial					0.24	0.07	3.95	0.000	1.53	0.14	0.43
Asthma					0.12	0.09	1.98	0.049	1.49	0.00	0.34
Age					0.56	0.32	8.99	0.000	1.59	2.23	3.49
Percent low income					0.07	3.45	1.02	0.307	2.01	−3.27	10.32
Population density					0.00	0.00	−0.05	0.963	2.31	0.00	0.00
Elevation					0.00	0.00	0.00	0.998	2.10	0.00	0.00

**Table 4:** Multiple regression models for interactions predicting depressive symptoms.

Predictor	R	R^2^	F	Sig.	*b*	*SE*	*t*	*p*	VIF	95% CI	
Model Gender × Depressive Sx	0.67	0.45	11.37	0.000							
Ozone					0.19	0.39	2.04	0.043	3.43	0.03	1.57
Male					−0.09	0.07	−1.61	0.108	1.10	−0.24	0.02
Male × Ozone					−0.13	0.04	−2.25	0.026	1.18	−0.17	−0.01
American Indian or Alaskan Native					0.10	0.24	1.71	0.088	1.26	−0.06	0.87
African American					0.05	0.08	0.80	0.423	1.44	−0.09	−0.22
Hispanic					0.08	0.03	1.34	0.181	1.45	−0.02	0.08
Asian					−0.09	0.23	−1.26	0.208	1.70	−0.73	0.16
Middle Eastern, North African, or Arab					−0.03	0.75	−0.40	0.692	1.61	−1.77	1.18
Native Hawaiian or Pacific Islander					−0.09	1.06	−1.65	0.100	1.16	−3.84	0.34
Multiracial					0.34	0.08	5.33	0.000	1.53	0.26	0.56
Asthma					0.11	0.09	1.74	0.083	1.61	−0.02	0.34
Age					0.45	0.31	7.39	0.000	1.41	1.70	2.94
Percent low income					0.13	3.36	1.89	0.060	1.80	−0.26	12.98
Population density					0.04	0.00	0.64	0.524	1.74	0.00	0.00
Elevation					−0.09	0.00	−0.93	0.355	3.86	0.00	0.00
Model Race × Depressive Sx	0.69	0.47	18.98	0.000							
Ozone					0.18	0.37	1.95	0.053	3.30	−0.01	1.46
Nonwhite					0.30	0.02	4.76	0.000	1.60	0.06	0.14
Nonwhite × Ozone					0.11	0.01	2.06	0.041	1.13	0.00	0.04
Nonbinary					0.35	0.14	5.78	0.000	1.46	0.53	1.07
Female					0.00	0.06	0.05	0.962	1.21	−0.12	0.13
Asthma					0.19	0.08	3.22	0.002	1.34	0.10	0.42
Age					0.52	0.31	8.59	0.000	1.45	2.06	3.27
Percent low income					0.18	2.88	3.03	0.003	1.41	3.04	14.40
Population density					−0.12	0.00	−1.61	0.109	1.76	0.00	0.00
Elevation					−0.12	0.00	−1.12	0.264	3.60	−0.004	0.00
Model Age × Depressive Sx	0.71	0.51	13.46	0.000							
Ozone					0.18	0.38	1.97	0.050	3.54	0.00	1.49
Age					0.55	0.32	9.03	0.000	1.59	2.23	3.47
Age × Ozone					0.04	0.20	0.73	0.467	1.14	−0.25	0.54
Nonbinary					0.33	0.14	5.51	0.000	1.55	0.49	1.04
Female					0.02	0.07	0.39	0.701	1.37	−1.05	0.16
American Indian or Alaskan Native					0.12	0.22	2.12	0.035	1.26	0.03	0.91
African American					0.05	0.08	0.89	0.377	1.45	−0.08	0.21
Hispanic					0.20	0.03	3.24	0.001	1.63	0.03	0.13
Asian					−0.08	0.21	−1.31	0.193	1.70	−0.70	0.14
Middle Eastern, North African, or Arab					0.00	0.71	−0.01	0.996	1.62	−1.40	1.39
Native Hawaiian or Pacific Islander					−0.12	1.00	−2.21	0.028	1.16	−4.19	−0.24
Multiracial					0.24	0.07	4.07	0.000	1.51	0.15	0.43
Asthma					0.12	0.09	2.03	0.044	1.51	0.01	0.34
Percent low income					0.13	3.18	1.97	0.050	1.80	0.00	12.52
Population density					−0.06	0.00	−0.93	0.353	1.83	0.00	0.00
Elevation					−0.13	0.00	−1.32	0.188	3.89	−0.01	0.00

## Data Availability

The original data presented in the study were obtained from the Colorado Department of Public Health and Education (CDPHE) and are openly available at https://teeo-cdphe.shinyapps.io/COEnviroScreen_English/ (accessed on 15 March 2023) and https://cdphe.colorado.gov/healthy-kids-colorado-survey-dashboard/ (accessed on 16 March 2023). Materials and analysis code for this study can be shared upon request.

## References

[1] World Health Organization (2017). Depression and Other Common Mental Disorders Global Health Estimates.

[2] Proudman D., Greenberg P., Nellesen D. (2021). The Growing Burden of Major Depressive Disorders (MDD): Implications for Researchers and Policy Makers. Pharmacoeconomics.

[3] Wickersham A., Sugg H.V.R., Epstein S., Stewart R., Ford T., Downs J. (2021). Systematic Review and Meta-analysis: The Association Between Child and Adolescent Depression and Later Educational Attainment. J. Am. Acad. Child. Adolesc. Psychiatry.

[4] Jonsson U., Bohman H., Von Knorring L., Olsson G., Paaren A., Von Knorring A.L. (2011). Mental health outcome of long-term and episodic adolescent depression: 15-year follow-up of a community sample. J. Affect. Disord..

[5] Daly M. (2022). Prevalence of Depression Among Adolescents in the U.S. From 2009 to 2019: Analysis of Trends by Sex, Race/Ethnicity, and Income. J. Adolesc. Health.

[6] Keyes K.M., Gary D., O’Malley P.M., Hamilton A., Schulenberg J. (2019). Recent increases in depressive symptoms among US adolescents: Trends from 1991 to 2018. Soc. Psychiatry Psychiatr. Epidemiol..

[7] (2022). National Institute of Mental Health. https://www.nimh.nih.gov/health/statistics/major-depression.

[8] Schaakxs R., Comijs H.C., van der Mast R.C., Schoevers R.A., Beekman A.T.F., Penninx B.W.J.H. (2017). Risk Factors for Depression: Differential Across Age?. Am. J. Geriatr. Psychiatry.

[9] WHO (2022). Ambient (Outdoor) Air Pollution Fact Sheet. https://www.who.int/news-room/fact-sheets/detail/ambient-(outdoor)-air-quality-and-health.

[10] Environmental Protection Agency (2023). Our Nation’s Air. https://gispub.epa.gov/air/trendsreport/2023/#welcome.

[11] American Lung Association (2022). State of the Air. https://www.lung.org/research/sota/key-findings.

[12] Landrigan P.J. (2017). Air pollution and health. Lancet Public Health.

[13] Chen H., Goldberg M.S., Viileneuve P.J. (2008). A systematic review of the relation between long-term exposure to ambient air pollution and chronic diseases. Rev. Environ. Health.

[14] Hoek G., Krishnan R.M., Beelen R., Peters A., Ostro B., Brunekreef B., Kaufman J.D. (2013). Long-term air pollution exposure and cardio-respiratory mortality: A review. Environ. Health.

[15] Al-Kindi S.G., Brook R.D., Biswal S., Rajagopalan S. (2020). Environmental determinants of cardiovascular disease: Lessons learned from air pollution. Nat. Rev. Cardiol..

[16] Fuhrmann D., Knoll L.J., Blakemore S.J. (2015). Adolescence as a Sensitive Period of Brain Development. Trends Cogn. Sci..

[17] Brockmeyer S., D’Angiulli A. (2016). How air pollution alters brain development: The role of neuroinflammation. Transl. Neurosci..

[18] Sacks J.D., Stanek L.W., Luben T.J., Johns D.O., Buckley B.J., Brown J.S., Ross M. (2011). Particulate matter-induced health effects: Who is susceptible?. Environ. Health Perspect..

[19] Borroni E., Pesatori A.C., Nosari G., Monti P., Ceresa A., Fedrizzi L., Bollati V., Buoli M., Carugno M. (2023). Understanding the Interplay between Air Pollution, Biological Variables, and Major Depressive Disorder: Rationale and Study Protocol of the DeprAir Study. Int. J. Environ. Res. Public Health.

[20] Arias-Pérez R.D., Taborda N.A., Gómez D.M., Narvaez J.F., Porras J., Hernandez J.C. (2020). Inflammatory effects of particulate matter air pollution. Environ. Sci. Pollut. Res..

[21] Yan Z., Jin Y., An Z., Liu Y., Samet J.M., Wu W. (2016). Inflammatory cell signaling following exposures to particulate matter and ozone. Biochim. Biophys. Acta (BBA)—Gen. Subj..

[22] Mumby S., Chung K.F., Adcock I.M. (2019). Transcriptional effects of ozone and impact on airway inflammation. Front. Immunol..

[23] Patel A. (2013). Review: The role of inflammation in depression. Psychiatr. Danub..

[24] Vert C., Sánchez-Benavides G., Martínez D., Gotsens X., Gramunt N., Cirach M., Molinuevo J.L., Sunyer J., Nieuwenhuijsen M.J., Crous-Bou M. (2017). Effect of long-term exposure to air pollution on anxiety and depression in adults: A cross-sectional study. Int. J. Hyg. Environ. Health.

[25] Xue T., Guan T., Zheng Y., Geng G., Zhang Q., Yao Y., Zhu T. (2021). Long-term PM2.5 exposure and depressive symptoms in China: A quasi-experimental study. Lancet Reg. Health West. Pac..

[26] He G., Chen Y., Wang S., Dong Y., Ju G., Chen B. (2020). The Association Between PM2.5 and Depression in China. Dose-Response.

[27] Zhao T., Markevych I., Romanos M., Nowak D., Heinrich J. (2018). Ambient ozone exposure and mental health: A systematic review of epidemiological studies. Environ. Res..

[28] Lim Y.H., Kim H., Kim J.H., Bae S., Park H.Y., Hong Y.C. (2012). Air Pollution and Symptoms of Depression in Elderly Adults. Environ. Health Perspect..

[29] Gu X., Guo T., Si Y., Wang J., Zhang W., Deng F., Chen L., Wei C., Lin S., Guo X. (2020). Association between ambient air pollution and daily hospital admissions for depression in 75 Chinese cities. Am. J. Psychiatry.

[30] Zeng Y., Lin R., Liu L., Liu Y., Li Y. (2019). Ambient air pollution exposure and risk of depression: A systematic review and meta-analysis of observational studies. Psychiatry Res..

[31] Yolton K., Khoury J.C., Burkle J., LeMasters G., Cecil K., Ryan P. (2019). lifetime exposure to traffic-related air pollution and symptoms of depression and anxiety at age 12 years. Environ. Res..

[32] Latham R.M., Kieling C., Arseneault L., Rocha T.B.M., Beddows A., Beevers S.D., Danese A., De Oliveira K., Kohrt B.A., Moffitt T.E. (2021). Childhood exposure to ambient air pollution and predicting individual risk of depression onset in UK adolescents. J. Psychiatr. Res..

[33] Manczak E.M., Miller J.G., Gotlib I.H. (2022). Census tract ambient ozone predicts trajectories of depressive symptoms in adolescents. Dev. Psychol..

[34] Zhao T., Markevych I., Standl M., Schulte-Körne G., Schikowski T., Berdel D., Koletzko S., Bauer C.P., von Berg A., Nowak D. (2019). Ambient ozone exposure and depressive symptoms in adolescents: Results of the GINIplus and LISA birth cohorts. Environ. Res..

[35] Anderson E.R., Mayes L.C. (2010). Race/ethnicity and internalizing disorders in youth: A review. Clin. Psychol. Rev..

[36] Wilson S., Dumornay N.M. (2022). Rising Rates of Adolescent Depression in the United States: Challenges and Opportunities in the 2020s. J. Adolesc. Health.

[37] Jones M.R., Diez-Roux A.V., Hajat A., Kershaw K.N., O’Neill M.S., Guallar E., Post W.S., Kaufman J.D., Navas-Acien A. (2014). Race/ethnicity, residential segregation, and exposure to ambient air pollution: The Multi-Ethnic Study of Atherosclerosis (MESA). Am. J. Public. Health.

[38] Shorey S., Ng E.D., Wong C.H.J. (2022). Global prevalence of depression and elevated depressive symptoms among adolescents: A systematic review and meta-analysis. Br. J. Clin. Psychol..

[39] Ferlatte O., Salway T., Rice S.M., Oliffe J.L., Knight R., Ogrodniczuk J.S. (2020). Inequities in depression within a population of sexual and gender minorities. J. Ment. Health.

[40] Gridley S.J., Crouch J.M., Evans Y., Eng W., Antoon E., Lyapustina M., Schimmel-Bristow A., Woodward J., Dundon K., Schaff R. (2016). Youth and Caregiver Perspectives on Barriers to Gender-Affirming Health Care for Transgender Youth. J. Adolesc. Health.

[41] Lu W., Todhunter-Reid A., Mitsdarffer M.L., Muñoz-Laboy M., Yoon A.S., Xu L. (2021). Barriers and Facilitators for Mental Health Service Use Among Racial/Ethnic Minority Adolescents: A Systematic Review of Literature. Front. Public Health.

[42] Mital A. (2023). Change in environmental justice scores in historically redlined communities compared to non-redlined communities: A case study of Richmond, Virginia. Urban Clim..

[43] Geographic Products Branch U.S. Census Bureau Census Tracts. https://www2.census.gov/geo/pdfs/education/CensusTracts.pdf.

[44] Reddy P.J., Pfister G.G. (2016). Meteorological factors contributing to the interannual variability of midsummer surface ozone in Colorado, Utah, and other western U.S. states. J. Geophys. Res. Atmos..

[45] Chen W., Yan L., Zhao H. (2015). Seasonal Variations of Atmospheric Pollution and Air Quality in Beijing. Atmosphere.

[46] (2021). Healthy Kids Colorado Survey Dashboard|Department of Public Health & Environment. https://cdphe.colorado.gov/healthy-kids-colorado-survey-dashboard.

[47] Colorado Department of Public Health & Environment (2022). Colorado EnviroScreen. https://cdphe.colorado.gov/enviroscreen.

[48] United States Geological Survey (2023). USGS Data Releases. https://www.usgs.gov/products/data/data-releases.

[49] US Census Bureau (2022). American Community Survey. https://www.census.gov/programs-surveys/acs/about.html.

[50] America Counts Staff (2021). Colorado: 2020 Census. https://www.census.gov/library/stories/state-by-state/colorado-population-change-between-census-decade.html.

[51] Reff A., Philips S., Eyth A., Mintz D. (2020). Bayesian Space-Time Downscaling Fusion Model (Downscaler)-Derived Estimates of Air Quality for 2017. https://ofmpub.epa.gov/rsig/rsigserver?data/FAQSD/docs/2017_DS_Annual_Report.pdf.

[52] Colorado EnviroScreen Tool Team Denver, Colorado. 2023. Colorado EnviroScreen Tool Technical Document. https://drive.google.com/file/d/1aZfZnLeEPxvpFBILOFGpYGKLQbDxhMMF/view.

[53] Turon H., Carey M., Boyes A., Hobden B., Dilworth S., Sanson-Fisher R. (2019). Agreement between a single-item measure of anxiety and depression and the Hospital Anxiety and Depression Scale: A cross-sectional study. PLoS ONE.

[54] Zimmerman M., Ruggero C.J., Chelminski I., Young D., Posternak M.A., Friedman M., Boerescu D., Attiullah N. (2006). Developing Brief Scales for Use in Clinical Practice: The Reliability and Validity of Single-Item Self-Report Measures of Depression Symptom Severity, Psychosocial Impairment Due to Depression, and Quality of Life. J. Clin. Psychiatry..

[55] Colorado General Assembly 74th General Assembly. 2021. Environmental Justice Disproportionate Impacted Community. https://leg.colorado.gov/bills/hb21-1266.

[56] Lu Y., Mak K.K., van Bever H.P.S., Ng T.P., Mak A., Ho R.C.M. (2012). Prevalence of anxiety and depressive symptoms in adolescents with asthma: A meta-analysis and meta-regression. Pediatr. Allergy Immunol..

[57] Tiotiu A.I., Novakova P., Nedeva D., Chong-Neto H.J., Novakova S., Steiropoulos P., Kowal K. (2020). Impact of Air Pollution on Asthma Outcomes. Int. J. Environ. Res. Public Health.

[58] Gehring U., Wijga A.H., Koppelman G.H., Vonk J.M., Smit H.A., Brunekreef B. (2020). Air pollution and the development of asthma from birth until young adulthood. Eur. Respir. J..

[59] Semple J.L., Moore G.W.K. (2020). High levels of ambient ozone (O3) may impact COVID-19 in high altitude mountain environments. Respir. Physiol. Neurobiol..

[60] Hernández-Vásquez A., Vargas-Fernández R., Rojas-Roque C., Gamboa-Unsihuay J.E. (2022). Association between altitude and depression in Peru: An 8-year pooled analysis of population-based surveys. J. Affect. Disord..

[61] U.S. Geological Survey (2023). https://www.usgs.gov/educational-resources/highest-and-lowest-elevations.

[62] Borck R., Schrauth P. (2021). Population density and urban air quality. Reg. Sci. Urban. Econ..

[63] Rautio N., Filatova S., Lehtiniemi H., Miettunen J. (2018). Living environment and its relationship to depressive mood: A systematic review. Int. J. Soc. Psychiatry.

[64] IBM Corp (2021). IBM SPSS Statistics for Windows, Version 28.0. https://www.ibm.com/spss.

[65] González-Guevara E., Martínez-Lazcano J.C., Custodio V., Hernández-Cerón M., Rubio C., Paz C. (2014). Exposure to ozone induces a systemic inflammatory response: Possible source of the neurological alterations induced by this gas. Inhal. Toxicol..

[66] Velázquez-Pérez R., Rodríguez-Martínez E., Valdés-Fuentes M., Gelista-Herrera N., Gómez-Crisóstomo N., Rivas-Arancibia S. (2021). Oxidative Stress Caused by Ozone Exposure Induces Changes in P2 × 7 Receptors, Neuroinflammation, and Neurodegeneration in the Rat Hippocampus. Oxid. Med. Cell Longev..

[67] Ayeni E.A., Aldossary A.M., Ayejoto D.A., Gbadegesin L.A., Alshehri A.A., Alfassam H.A., Afewerky H.K., Almughem F.A., Bello S.M., Tawfik E.A. (2022). Neurodegenerative Diseases: Implications of Environmental and Climatic Influences on Neurotransmitters and Neuronal Hormones Activities. Int. J. Environ. Res. Public Health.

[68] Reuben A., Arseneault L., Beddows A., Beevers S.D., Moffitt T.E., Ambler A., Latham R.M., Newbury J.B., Odgers C.L., Schaefer J.D. (2021). Association of Air Pollution Exposure in Childhood and Adolescence With Psychopathology at the Transition to Adulthood. JAMA Netw. Open.

[69] Borroni E., Pesatori A.C., Bollati V., Buoli M., Carugno M. (2022). Air pollution exposure and depression: A comprehensive updated systematic review and meta-analysis. Environ. Pollut..

[70] Environmental Protection Agency (2023). Particulate Matter (PM2.5) Trends. https://www.epa.gov/air-trends/particulate-matter-pm25-trends.

[71] Brodin M., Helmig D., Oltmans S. (2010). Seasonal ozone behavior along an elevation gradient in the Colorado Front Range Mountains. Atmos. Environ..

[72] Urie Bronfenbrenner The Ecology of Human Development. 1979; Volume 7. https://books.google.com/books/about/The_Ecology_of_Human_Development.html?id=OCmbzWka6xUC.

[73] Environmental Protection Agency (2024). AirNow.gov. https://www.airnow.gov/?city=Denver&state=CO&country=USA.

[74] Environmental Protection Agency (2024). Overview of the Clean Air Act and Air Pollution. https://www.epa.gov/clean-air-act-overview.

[75] Jonidi Jafari A., Charkhloo E., Pasalari H. (2021). Urban air pollution control policies and strategies: A systematic review. J. Environ. Health Sci. Eng..

[76] Bien T., Helmig D. (2018). Changes in summertime ozone in Colorado during 2000–2015. Elementa.

[77] Xu L., Crounse J.D., Vasquez K.T., Allen H., Wennberg P.O., Bourgeois I., Brown S.S., Campuzano-Jost P., Coggon M.M., Crawford J.H. (2021). Ozone chemistry in western U.S. wildfire plumes. Sci. Adv..

